# Modeling the Effect of Hyperoxia on the Spin–Lattice Relaxation Rate R1 of Tissues

**DOI:** 10.1002/mrm.29315

**Published:** 2022-06-09

**Authors:** Emma Bluemke, Eleanor Stride, Daniel Peter Bulte

**Affiliations:** ^1^ Institute of Biomedical Engineering Department of Engineering Sciences, University of Oxford Oxford UK

**Keywords:** hyperoxia, longitudinal relaxation, oxygen, oxygen‐enhanced MRI, R1, tissue

## Abstract

**Purpose:**

Inducing hyperoxia in tissues is common practice in several areas of research, including oxygen‐enhanced MRI (OE‐MRI), which attempts to use the resulting signal changes to detect regions of tumor hypoxia or pulmonary disease. The linear relationship between PO_2_ and R1 has been reproduced in phantom solutions and body fluids such as vitreous fluid; however, in tissue and blood experiments, factors such as changes in deoxyhemoglobin levels can also affect the ΔR1.

**Theory and Methods:**

This manuscript proposes a three‐compartment model for estimating the hyperoxia‐induced changes in R1 of tissues depending on B0, SO_2_, blood volume, hematocrit, oxygen extraction fraction, and changes in blood and tissue PO_2_. The model contains two blood compartments (arterial and venous) and a tissue compartment. This model has been designed to be easy for researchers to tailor to their tissue of interest by substituting their preferred model for tissue oxygen diffusion and consumption. A specific application of the model is demonstrated by calculating the resulting ΔR1 expected in healthy, hypoxic and necrotic tumor tissues. In addition, the effect of sex‐based hematocrit differences on ΔR1 is assessed.

**Results:**

The ΔR1 values predicted by the model are consistent with reported literature OE‐MRI results: with larger positive changes in the vascular periphery than hypoxic and necrotic regions. The observed sex‐based differences in ΔR1 agree with findings by Kindvall et al. suggesting that differences in hematocrit levels may sometimes be a confounding factor in ΔR1.

**Conclusion:**

This model can be used to estimate the expected tissue ΔR1 in oxygen‐enhanced MRI experiments.

## Abstract 2

Click here for author‐reader discussions

## 1 INTRODUCTION

Many researchers have investigated using the paramagnetic relaxivity effect of oxygen on longitudinal relaxation rate R1 (1/T1) as a means of inferring oxygenation levels. For example, measurements of R1 have been used to infer oxygen levels in vitreous fluid as a noninvasive alternative to the highly invasive oxygen electrodes used to measure retinal hypoxia,^1^, ^2^, ^3^ bladder urine,^4^ and urine in the renal pelvis to create a non‐invasive detection of renal dysfunction,^5^ and cerebrospinal fluid.^4^, ^6^ Additionally, measuring changes in R1 following the inspiration of increased fractions of oxygen is the basis for oxygen‐enhanced MRI techniques,^7^, ^8^, ^9^ which are used to study a range of conditions from tumor hypoxia^8^, ^10^, ^11^ to lung disease.^12^, ^13^

The linear relationship between the partial pressure of oxygen (PO_2_) in a material and the resulting longitudinal relaxation rate (R1) has been measured in phantoms^2^, ^3^, ^4^, ^6^, ^14^ and bodily fluids such as vitreous fluid.^2^ The relationship between R1 and PO_2_ has been modeled as for a paramagnetic contrast agent, R1_Ox_ = R1_0_ + r1_Ox_*C, where R1_Ox_ is the relaxation rate in the solution with oxygen added, R1_0_ is the relaxation rate in the solution without oxygen, C is the concentration of oxygen added, and r1_Ox_ is the relaxivity of oxygen in that solution, which is dependent on the magnetic field and temperature.^15^ Although this linear relationship has been demonstrated in phantoms and bodily fluids, studies on blood and tissues have sometimes reported so‐called ”contradictory” R1 changes, where either no change or a negative change in R1 is observed.^16^, ^17^ It has been hypothesized that the source of this contradictory R1 change is paramagnetic deoxyhemoglobin since there is a positive linear relationship between R1 and deoxyhemoglobin concentration, i.e., an inverse correlation with blood oxygen saturation.^18^ The blood oxygen saturation, denoted by ‘SO_2_’, is a measure of how much hemoglobin is currently bound to oxygen compared to how much hemoglobin remains unbound. In contrast, the partial pressure of oxygen (PO_2_) in blood is a measure of the dissolved oxygen in the plasma.

To estimate changes in blood R1 following hyperoxia, Bluemke et al.^19^ created a general model to estimate the R1 of blood, accounting for hematocrit, oxygen saturation (SO_2_), the partial pressure of oxygen (PO_2_), and magnetic field strength under both normal physiological and hyperoxic conditions. That model showed that there are two competing effects on blood R1 that arise from increasing oxygen levels (paramagnetic oxygen and paramagnetic deoxyhemoglobin) and that the effect on R1 due to deoxyhemoglobin dominates at SO_2_ levels below 99%, thus inducing a negative ΔR1 in venous blood after breathing 100% oxygen. While this model does explain the negative ΔR1 measured by Vatnehol et al. in venous blood during oxygen delivery experiment,^16^ it is not directly applicable to applications such as placental and tumor OE‐MRI research where image voxels contain non‐vascular tissue. In a tissue voxel, changing deoxyhemoglobin levels will only affect the portion of the voxel that is occupied by blood, and the R1 change of the tissue will dominate the remaining voxel volume. Therefore, in order to estimate the expected change in R1 of a tissue voxel, the blood model^19^ must be extended to contain a tissue compartment.

In this paper, we present a three‐compartment model for estimating the changes in R1 that could be expected in healthy, hypoxic, and necrotic tissues depending on field strength, blood oxygen saturation, blood volume, hematocrit, oxygen extraction fraction, and change in partial pressure of oxygen. Since modeling tissue oxygen diffusion and consumption is a broad, active research area with many different approaches, this model has been designed to make it possible for a researcher to easily substitute their preferred model for tissue oxygen diffusion and consumption and tailor this model to their tissue of interest. For this paper, the model incorporates the classic Krogh tissue cylinder model for oxygen diffusion and the commonly used Michaelis–Menten equation for oxygen consumption. Last, we demonstrate the use of this model for estimating the expected R1 changes in tissues from breathing increased levels of oxygen and compare the R1 estimations with literature empirical measurements from oxygen‐enhanced MRI research.^8^, ^10^, ^11^, ^20^, ^21^

## 2 THEORY

### 2.1 Model background and overview

There have been two previous attempts at modeling the changes in tissue R1 following increased oxygen: both arise from the research field of OE‐MRI.^22^, ^23^ In 2013, Holliday^22^ used a two‐compartment model of blood to estimate ΔR1 in a capillary given a change in capillary PO_2_ (Equation 1) and used a Krogh tissue cylinder model to estimate the corresponding tissue ΔPO_2_ (ΔPO_2T_), allowing the ΔR1 in tissue to be calculated by Equation (2).

(1) $$\Delta R1_{\text{capillary}}=\Delta PO_{2}*r1_{\mathrm{Ox}}+\Delta \left(1-SO_{2}\right)*r1_{\mathrm{dHb}}$$

(2) $$\Delta R1_{tis}=\Delta PO_{2\;\mathrm{tis}}*r1_{\mathrm{Ox}}\;.$$

Holliday used the r1_dHb_ at 1.5T reported by Blockley et al. (0.11 s^−1^).^18^ The main limitation of Holliday's approach is that the resulting model contains many independently adjustable variables, such as the blood flow velocity, oxygen consumption rate, vessel geometry, and length of the capillary. In practice, these variables are often impossible to measure in tissues.

In 2018, Kindvall^23^ proposed a much simpler equation to estimate the expected R1 change in pulmonary OE‐MRI, in which the total ΔR1 is separated into the expected ΔR1 of the arterial blood (ΔR1_B,A_), venous blood (ΔR1_B,V_), and tissue ΔR1_T_, and divided by 3 to yield an average ΔR1:

(3) $$\Delta R1_{\mathrm{tis}}=\frac{\Delta R1_{B,A}+\Delta R1_{B,V}+\Delta R1_{T}}{3\;}\;.$$

Kindvall then used empirical measurements from previous experiments to estimate ΔR1 at a set magnetic field strength (4.7T): the ΔR1_B,A_ and ΔR1_T_ were set to be the same values (0.2 s^−1^), and ΔR1_B,V_ was estimated to be −0.05 s^−1^ based on the relaxivity of deoxyhemoglobin (r1_dHb_) reported by Silvennoinen et al. (0.35 s^−1^) and a rough estimate of the expected change in venous deoxyhemoglobin levels.^24^ The main insight provided by Kindvall's approach was the benefit of separating the R1 changes in arterial blood and venous blood to observe the negative ΔR1 induced in deoxygenated blood, as opposed to Holliday's approach of estimating the ΔR1 of the capillary blood. However, the division by 3 assumes a 66% blood volume fraction in the voxel, which is too high for most tissue voxels.

Last, some substantial limitations to both of these approaches are: (A) the equations used to calculate the blood ΔR1 were of unknown accuracy and were not compared with literature values, (B) they did not take into account the blood hematocrit levels, and (C) the equations were not adjustable for magnetic field strength, which has a considerable effect on the relaxivity of oxygen.^15^

Therefore, we propose a new three‐compartment model that calculates the expected ΔR1 of the arterial blood (ΔR1_B,A_), venous blood (ΔR1_B,V_), and tissue ΔR1_T_ as separate compartments, and, following Holliday, uses a Krogh tissue cylinder model to estimate the corresponding tissue ΔPO_2_ (ΔPO_2T_). The model takes into account the magnetic field strength (B0), blood SO_2_, blood volume fraction (BV), hematocrit (Hct), oxygen extraction fraction of the tissue (OEF), and changes in PO_2_ in both the blood and tissue. Last, the model uses the blood volume fraction to calculate the resulting ΔR1 of the voxel (ΔR1_voxel_).

**FIGURE 1:**
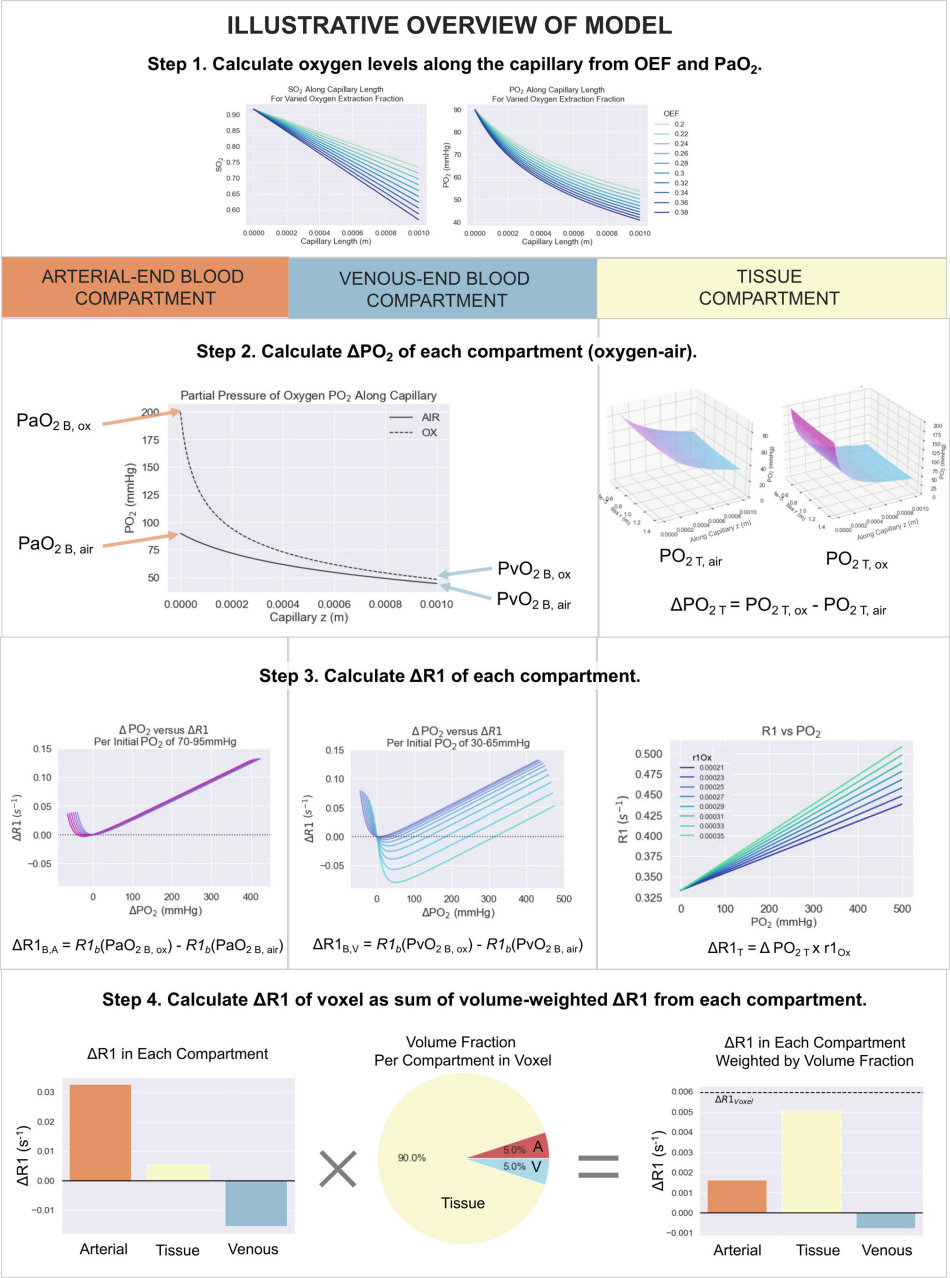
An illustrative overview of the three‐compartment model, which can be separated conceptually into four steps as shown. The inputs and outputs of the functions used in each step are shown in Figure 2

An overview of the model is illustrated in Figure 1. The model can be conceptually separated into four steps:

Step 1. Calculate PO_2_ along the capillary. The ΔR1_B,V_ and ΔR1_T_ and ΔR1_B,A_ are all related by a calculation of the SO_2_ or PO_2_ along the capillary length, which is determined by the arterial PO_2_ (PaO_2_) and the OEF of the tissue.
Step 2. Calculate ΔPO_2_ of each compartment. Knowing the PO_2_ along the capillary allows the ΔPO_2_ from oxygen administration to be calculated in the blood and tissue compartments, using the Krogh tissue cylinder to model the oxygen diffusion into the tissue, and the set OEF is related to the tissue oxygen consumption rate. The Krogh tissue cylinder radius is calculated from the set blood volume.
Step 3. Calculate ΔR1 of each compartment. Knowing the ΔPO_2_ in each compartment allows: (A) the ΔR1_T_ to be calculated using the relaxivity of oxygen (r1_Ox_) as a function of the magnetic field, using the equation for r1_Ox_ by Bluemke et al.^15^; and (B) the ΔR1 of each blood compartment to be calculated using the Blood R1 model published by Bluemke et al.^19^
Step 4. Calculate ΔR1 of the voxel. Once the ΔR1 in each compartment is calculated, the set blood volume fraction (BV) is used to calculate the resulting ΔR1 of the voxel (ΔR1_voxel_).

**FIGURE 2:**
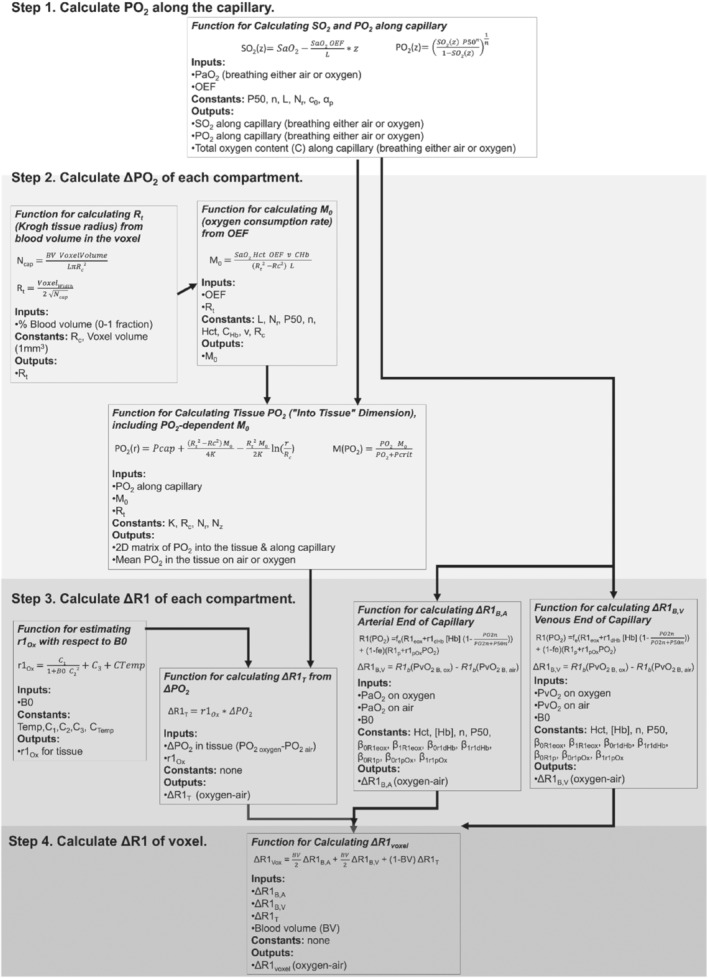
The inputs and outputs of each resulting function used in this model

The theory and reasoning behind each part of this model are provided in the following sections. The model has been provided as a public code repository, and a graphical overview of the inputs and outputs of each function created to implement this model is provided in Figure 1. This model was created as a set of Python functions, the overview of which is provided in Figure 2.

### 2.2 Step 1: calculating PO_2_ along capillary length

In this step, the PO_2_ along the capillary is calculated using arterial oxygen content and oxygen extraction fraction inputs. The output of this step is the known SO_2_ and PO_2_ along the capillary, which can be calculated before and after the oxygen delivery or hyperoxic gas challenge.

The total concentration of oxygen (c) in the blood is the sum of the concentrations of bound and dissolved oxygen^25^:

(4) $$c=c_{\text{dissolved}}+c_{\text{bound}}=\alpha_{p}*PO_{2}+\mathrm{Hct}*c_{0}*SO_{2},$$

where *c* is the total blood oxygen concentration, and c_0_ is the concentration of oxygen per unit volume of red blood cells (RBCs) at maximal saturation, and a_p_ is the solubility of oxygen in plasma assuming 22.4 L/mol under normal conditions.^25^ The total concentration (c) can also be referred to as the arterial oxygen content (C_a_O_2_) and venous oxygen content (C_v_O_2_). The following relationship between C_a_O_2_ and C_v_O_2_ defines the oxygen extraction fraction (OEF), which is the ratio of blood oxygen that tissue takes from the blood flow^26^:

(5) $$\mathrm{OEF}=\frac{C_{a}O_{2}-C_{v}O_{2}}{C_{a}O_{2}}.$$

Using developed formalism from Gjedde et al.^27^ and Rasmussen et al.,^28^ assuming that the total oxygen extraction rises linearly with distance along the capillary, as established by Kety et al.,^29^ the mean SO_2_ in the capillary (SO_2 cap,mean_) can be defined in relation to OEF as^26^:

(6) $$SO_{2_{\mathrm{cap},\text{mean}}}=\mathrm{Sa}O_{2}\left(1-\frac{\mathrm{OEF}}{2}\right).$$

The total oxygen content along the capillary can therefore be calculated for any given OEF (shown in Supporting Information Figure S1, which is available online) and initial SaO_2_, PaO_2_, or CaO_2_ value, and find the resulting PO_2_ and SO_2_ along the capillary length by using Equation (6) and the Hill equation^30^:

(7) $$SO_{2}\left(z\right)=\mathrm{Sa}O_{2}-\frac{\mathrm{Sa}O_{2}\;\mathrm{OEF}}{L}*z$$

(8) $$PO_{2}\left(z\right)={\left(\frac{SO_{2}\left(z\right)\;P50^{n}}{1-SO_{2}\left(z\right)}\right)}^{\left(1/n\right)}\;,$$

where P50 is the oxygen tension when hemoglobin is 50% saturated with oxygen, and n is the Hill exponent for hemoglobin (typically 2.7).^30^

### 2.3 Step 2: Estimating ΔPO_2_ in the three compartments

#### 2.3.1 Blood compartments

In this model, the arterial PO_2_ levels are chosen by the user. The arterial PO_2_ following a hyperoxic gas challenge such as is used in OE‐MRI, is estimated to increase from 90 mmHg to 600 mmHg based on empirical measurements,^31^ and therefore this is used for the duration of this paper. The corresponding change in venous PO_2_ will depend on the OEF, and is therefore calculated by the PO_2_ values at the venous end of the capillary at baseline or with supplemental oxygen.

#### 2.3.2 Tissue compartment

##### Model for tissue oxygen diffusion

A wide variety of models for tissue oxygen diffusion and metabolism have been established, accounting for differing capillary networks, geometry, and other special features. For the purpose of this paper, we use the classic Krogh tissue cylinder model and common Michaelis–Menten equation for oxygen consumption rate, however any preferred tissue model can be substituted to calculate the resulting change in tissue oxygen levels (ΔPO_2T_) from hyperoxia to use in Step 3. The Krogh‐Erlang solution has the following assumptions, paraphrased from Goldman et al.^30^: (A) tissue oxygen consumption is constant and uniform; (B) tissue oxygen at the capillary wall equals average capillary PO_2_; (C) tissue oxygen solubility and diffusivity are uniform (D) axial (or longitudinal) diffusion of oxygen is not significant; (E) all important microvascular oxygen transport phenomena are steady‐state; (F) all capillaries are parallel, unbranched, and equally spaced; (G) all capillaries receive equal convective oxygen supply; (H) capillaries are the only microvessels that play a role in oxygen transport to tissue.

For steady state PO_2_ in the tissue cylinder and a given capillary PO_2_, the Krogh‐Erlang solution is:

(9) $$PO_{2}\left(r\right)=P_{\mathrm{cap}}+\frac{M_{0}}{4K}\;\left(r^{2}-{R_{c}}^{2}\right)-\frac{M_{0}{R_{t}}^{2}}{2K}\;\ln\left(\frac{r}{R_{c}}\right)$$

where PO_2_(r) is the PO_2_ at r in the tissue, R_c_ is capillary radius, R_t_ is the Krogh tissue radius, P_cap_ is the PO_2_(R_c_) (partial pressure of oxygen at the capillary radius), r is the radial coordinate, M_0_ is the maximum tissue oxygen consumption rate, and K is the Krogh diffusion constant K=Da_T_, where D is the tissue oxygen diffusivity and a_T_ is the tissue oxygen solubility.^30^ The behavior of Equation (9) for different levels of P_cap_ is shown in Supporting Information Figure S2A.

##### Model for tissue oxygen consumption

As discussed by Goldman et al.,^30^ one important modification commonly made to the Krogh cylinder model is the addition of PO_2_‐dependent tissue oxygen consumption. The most common model, Michaelis–Menten, calculates the oxygen consumption rate by the following equation:

(10) $$M\left(PO_{2}\right)=\frac{M_{0}\;PO_{2}}{PO_{2}+P_{\text{crit}}}$$

where tissue oxygen consumption rate M(PO_2_) is found to be approximately constant for tissue PO_2_ above a certain value (P_crit_), and below this value, the oxygen consumption drops off sharply to zero.^30^ The behavior of Equation (10), and the effect of varying P_crit_, is shown in Supporting Information Figure S2B. Therefore, in this manuscript, the M_0_ constant in Equation (7) has been replaced with the function M(PO_2_) (Equation 10).

##### Estimating R_t_ from blood volume

Equation (9) requires knowledge of the Krogh tissue radius (R_t_), which can be estimated per the chosen blood volume (BV). Based on the Krogh assumptions above, we can model the capillaries as cylinders that are parallel, unbranched, and equally spaced throughout the 3D voxel (illustrated in Supporting Information Figure S3). Capillary length can range from 0.5–1.5 mm, so for simplicity in modeling a 1 mm^3^ voxel, we will assume a capillary length of 1 mm. The volume of one cylindrical capillary is:

(11) $$V_{\mathrm{cap}}=\pi {R_{c}}^{2}L.$$

The estimated blood volume can be related to the capillary density in the voxel through the following equation from which N_cap_ can be calculated (assuming a constant R_c_ and L):

(12) $$\mathrm{BV}=\frac{N_{\mathrm{cap}}xV_{\mathrm{cap}}}{V_{\text{voxel}}}.$$

In a cross‐section, based on the sixth assumption from the Krogh‐Erlang solution, the number of capillaries (N_cap_) are spaced evenly across the width and length of the voxel cross‐section. Therefore, R_t_ can be found by using the following equation:

(13) $$\text{Voxe}l_{\text{width}}=2R_{t}*\sqrt{N_{\mathrm{cap}}}.$$

##### Estimating Oxygen Consumption Rate M_0_ from OEF and R_t_

Using the Krogh‐Erlang solution (Equation 9) also requires knowledge of the maximum tissue oxygen consumption rate, M_0_. Once R_t_ is found, we can derive M_0_ from the OEF using the following logic and assumptions. It is important to note that in order for the assumptions of the following equations to be valid, M_0_ must be calculated in the normoxic state (i.e., the patient is breathing air). Under this assumption, the SO_2_ along the capillary can be calculated using the linear equation^30^:

(14) $$SO_{2}\left(z\right)=\mathrm{Sa}O_{2}-\frac{\left({R_{t}}^{2}-{R_{c}}^{2}\right)\;M_{0}}{\mathrm{Hct}\;C_{\mathrm{Hb}}\;v}*z.$$

Using Equation (6), which relates mean SO_2_ to the OEF, we can create a linear equation of SO_2_ along z, the mean SO_2_ in the capillary (SO_2 cap,mean_) will be equal to the linear Equation (14) at 0.5 L. Therefore, M_0_ can be related to OEF using the following steps:

(15) $$\begin{array}{cc} SO_{2_{\mathrm{cap},\text{mean}}} & =\mathrm{Sa}O_{2}*\left(1-\frac{\mathrm{OEF}}{2}\right)=SO_{2}\left(0.5L\right)\\ & =\mathrm{Sa}O_{2}-\frac{\left({R_{t}}^{2}-{R_{c}}^{2}\right)\;M_{0}}{\mathrm{Hct}\;\;C_{\mathrm{Hb}}\;v}*0.5L \end{array}$$

which can be rearranged to calculate M_0_:

(16) $$M_{0}=\frac{\mathrm{OEF}\;\mathrm{Hct}\;C_{\mathrm{Hb}}\;v\;\mathrm{Sa}O_{2}}{\left({R_{t}}^{2}-{R_{c}}^{2}\right)\;L}.$$

Finally, with M_0_ and R_t_ estimated from the OEF and blood volume, the tissue PO_2_ (into‐tissue axis) can be calculated. Since the PO_2_ along the capillary (P_cap_) on air and oxygen breathing is known from Step 1, the PO_2_ throughout the Krogh cylinder can be calculated for both normoxic and hyperoxic situations (shown in Supporting Information Figure S4).

### 2.4 Step 3. Calculating ΔR1 in the three compartments

#### 2.4.1 Blood compartments

**FIGURE 3:**
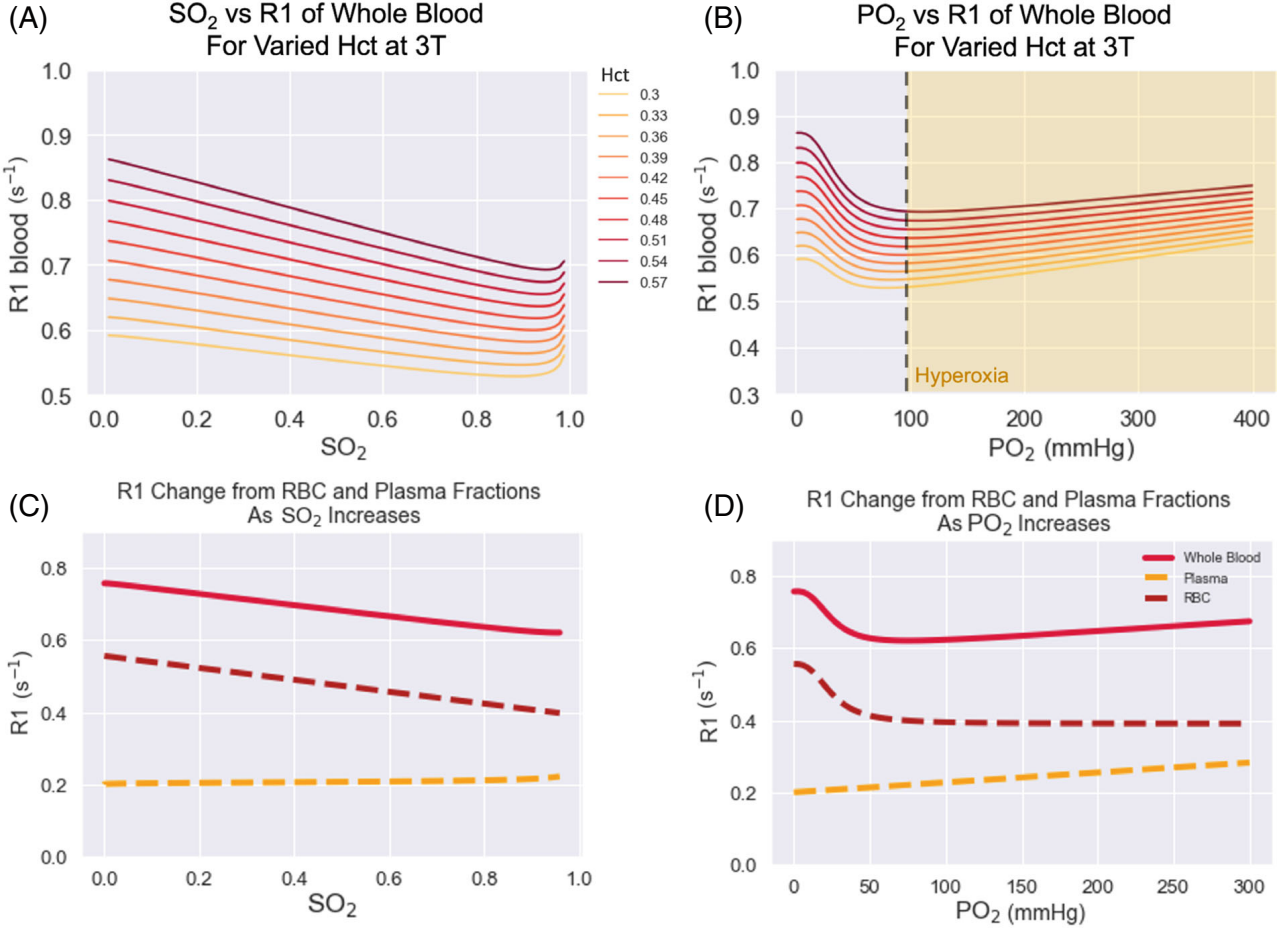
Plots with simulated data to illustrate the behavior of the model published by Bluemke et al., showing PO_2_ vs R1 (A) and SO_2_ vs R1 (B) for a range of hematocrit values (0.3–0.57) at 3T. To show the behavior of the two compartments of the model, the plasma fraction (yellow) and erythrocyte fraction (maroon) are shown separately as SO_2_ increases (C) and PO_2_ increases (D), with B0 = 3T, Hct = 0.42

The ΔR1 induced from supplemental oxygen in the arterial (ΔR1_B,A_) and venous (ΔR1_B,V_) blood compartments can both be calculated from the following equation

(17) $$\Delta R1_{B}=R1_{b}\left(PO_{2,\mathrm{ox}}\right)-R1_{b}\left(PO_{2,\mathrm{air}}\right)$$

where R1_b_(PO_2_) is the general equation for calculating the R1 of blood by Bluemke et al.^19^ (shown in Figure 3):

(18) $$\begin{array}{cc} R1_{b}\left(PO_{2}\right) & =f_{e}\left(R1_{\mathrm{eox}}+r1_{\mathrm{dHb}}\left[\mathrm{Hb}\right]\left(1-\frac{P{O_{2}}^{n}}{P{O_{2}}^{n}+P50^{n}}\right)\right)\\ & \;+\left(1-f_{e}\right)\left(R1_{p}+r1_{\mathrm{pOx}}PO_{2}\right) \end{array}$$

where R1_b_ is the relaxation rate of whole blood, R1_eox_ is the relaxation rate of erythrocytes when SO_2_ = 100%, [Hb] is the mean corpuscular hemoglobin concentration (5.15 mmol Hb tetramer/L plasma), r1_dHb_ is the molar relaxivity of deoxyhemoglobin (in s^−1^ L plasma in erythrocyte/mmol Hb tetramer), n is the Hill exponent for hemoglobin (typically 2.7),^30^ R1_p_ is the longitudinal relaxation rate of plasma (s^−1^), and r1_pOx_ is the relaxivity of dissolved oxygen in the plasma in s^−1^ mmHg^−1^ oxygen. The variable f_e_ is the fraction of water in whole blood that resides in erythrocytes (0–1), which is described by the equation:

(19) $$f_{e}\left(\mathrm{Hct}\right)=\frac{W_{\mathrm{RBC}}\mathrm{Hct}}{W_{\mathrm{RBC}}\mathrm{Hct}+W_{\text{plasma}}\left(1-\mathrm{Hct}\right)}$$

where Hct is the hematocrit (0–1), W_RBC_ is the volume fraction of water within the erythrocyte (typically valued at 0.70 due to hemoglobin occupying approximately 30% of the erythrocyte volume), and W_plasma_ is the volume fraction of water within the plasma (typically valued at 0.95, where the other 5% volume is occupied by plasma proteins such as albumin).^32^ In addition, Bluemke et al.^19^ modeled R1_eox_, R1_p_, r1_dHb_, and r1_pOx_ to have a linear dependence on B0 (where β_0_ and β_1_ are the y‐intercept and slope for the linear fit):

(20) $$R1_{\mathrm{eox}}\left(B0\right)=\beta_{0,R1_{\mathrm{eox}}}+\beta_{1,R1_{\mathrm{eox}}}B0$$

(21) $$R1_{p}\left(B0\right)=\beta_{0,R1_{p}}+\beta_{1,R1_{p}}B0$$

(22) $$r1_{\mathrm{dHb}}\left(B0\right)=\beta_{0,r1_{\mathrm{dHb}}}+\beta_{1,r1_{\mathrm{dHb}}}B0$$

(23) $$r1_{\mathrm{pOx}}\left(B0\right)=\beta_{0,r1_{\mathrm{pOx}}}+\beta_{1,r1_{\text{pOxB}0}}\;B0\;.$$

The values used for all empirically derived parameters and constants in the blood model are listed in the original publication.^19^ The behavior of this model is shown in Figure 3.

#### 2.4.2 Tissue compartment

**FIGURE 4:**
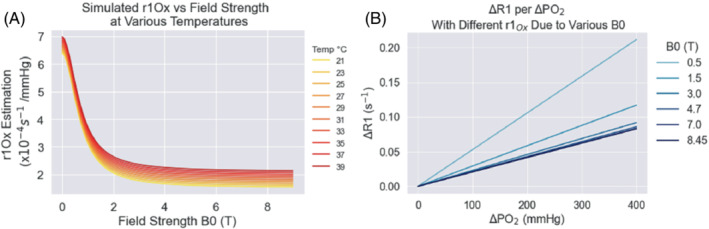
Simulated data to illustrate the effect of field strength on the relaxivity of oxygen (r1_Ox_) according to the model by Bluemke et al. (for a variety of temperatures) (A), and the resulting effect on R1 from different strengths of r1_Ox_ within a range of commonly used main magnetic fields (B0) (B)

Between the air and oxygen states, the only paramagnetic factor changing in the tissue compartment is the concentration of oxygen dissolved either intracellularly or interstitially. Therefore, the ΔPO_2T_ calculated in Step 2 can be used to calculate the ΔR1_T_ using the following equation

(24) $$\Delta R1_{T}=r1_{\mathrm{Ox}}*\Delta PO_{2\;T}$$

where the relaxivity of oxygen, r1_Ox_, is dependent on field strength and calculated using the empirical model for r1_Ox_ published by Bluemke et al.^15^ (shown in Figure 4):

(25) $$\;r1_{\mathrm{Ox}}=\frac{C_{1}}{1+C_{2}B0^{2}\;}+C_{3}+C_{\text{Temp}}*T$$

where the empirically derived constants are set to the parameters reported by Bluemke et al.,^15^ and in the context of this tissue model, temperature is assumed to be 37°C.

### 2.5 Step 4. Calculating weighted voxel ΔR1

Last, the model uses the blood volume fraction (BV) to calculate the resulting ΔR1 of the voxel (ΔR1_voxel_), assuming equal fractions of arterial and venous blood are present in the voxel, using the following equation:

(26) $$\Delta R1_{\text{voxel}}=\left(\;\frac{\mathrm{BV}}{2}\;\right)\;\Delta R1_{B,A}+\left(1-\mathrm{BV}\right)\Delta R1_{T}+\;\left(\;\frac{\mathrm{BV}}{2}\;\right)\;\Delta R1_{B,V}\;.$$

By keeping the arterial and venous changes separate, the +ΔR1 in more oxygenated blood will be accounted for in the “arterial end” compartment and the −ΔR1 in the less oxygenated blood will be accounted for in the “venous end” section. Ultimately, the large volume fraction of tissue contributes the most to the final ΔR1_voxel_ (see schematic in Supporting Information Figure S5).

## 3 METHODS

### 3.1 Applying model to simulate ΔR1 from OE‐MRI

#### 3.1.1 Model variables and constants

To demonstrate the application of this model, the five independent variables that vary between different types of tumor tissues, patients and experiments were: the magnetic field B0, hematocrit, oxygen extraction fraction, blood volume in the voxel, and P_crit_ — the ranges of these values used to produce the R1 estimates are listed in Table 1. Next, there are four dependent variables that are calculated from the independent variables: the fraction of erythrocytes in whole blood f_e_ is calculated from hematocrit; the r1_Ox_ is calculated from B0; the Krogh tissue radius R_t_ is calculated from OEF and blood volume, and the tissue oxygen consumption rate is calculated from OEF, R_t_, and P_crit_ (shown in Table 2). Last, the constants used for the remaining parameters were sourced from the literature and listed in Table 3.^25^, ^30^, ^33^, ^34^

**TABLE 1:** Five independent variables that vary between different types of tumor tissues, patients, and experiments

Variable Meaning	Variable Name	Healthy Brain Tissue	Tumor Vascular Periphery *No Tissue Compartment*	Tumor Tissue with High Metabolism i.e., Hypoxic *2 types: Low BV and High BV*	Tumor Tissue with Normal Metabolism	Tumor Necrotic Tissue *No Blood Compartments*	Units
Main magnetic field of MRI	B0	1.5, 3, 4.7, 7					T
Hematocrit	Hct	Total range: 0.36–0.50					0–1
		Female range: 0.36–0.48					Volume fraction
		Male range: 0.41–0.50					
Oxygen extraction fraction	OEF	0.31–0.37	0.24–0.44		0.02–0.2	0.02–0.05	0–1 Fraction
Blood volume in voxel	BV	0.02–0.07	1.0 (100% blood volume, no tissue compartment)	Low BV range = 0.047–0.059	0.047–0.059	0.001 (0.1% blood volume, i.e., no blood compartment)	0–1 Volume fraction
				High BV range = 0.09–0.17			
The PO_2_ value above which the tissue oxygen consumption rate is relatively constant	P_crit_	1–4	N/A	0.5–2			mmHg

*Note*: The chosen ranges of these values used to produce the simulated results in this paper are listed.

**TABLE 2:** Four dependent variables that are calculated from the independent variables listed in Table 1

Parameter meaning	Parameter name	Value or range used	Units
Fraction of erythrocytes in whole blood	f_e_	Calculated by Equation (19) as a function of Hct, W_RBC_ and W_plasma_	0–1 Volume fraction
Relaxivity of oxygen	r1_Ox_	Calculated by Equation (25) as a function of B0 and temperature (set to 37 °C for tissue model)	s^−1^ mmHg^−1^
Krogh tissue radius	R_t_	Determined by the blood volume	m
Tissue oxygen consumption	M_0_	Determined by the OEF and the R_t_	mlO_2_ ml^−1^ s^−1^

**TABLE 3:** Constants used for the remaining parameters of the model

Constant meaning	Name	Value used (units)	Source
Oxygen tension when hemoglobin is 50% saturated with oxygen	P50	37 (mmHg)	Goldman 2008
Hill coefficient	n	2.7 (unitless)	Goldman 2008
Plasma O_2_ solubility	a_p_	3.1 × 10^−5^ (mlO_2_ ml^−1^ mmHg^−1^)	Welter 2016
		*assuming 22.4 L/mol under normal conditions	
Hemoglobin binding capacity (Hüffner factor)	C_Hb_	1.36 (mlO_2_ g^−1^)	Welter 2016
Concentration of oxygen per unit volume of RBCs at maximal saturation: calculated as the product of the hemoglobin binding capacity C_Hb_ and the mean corpuscular hemoglobin concentration [Hb]	c_0_	Calculated from	Welter 2016
		c_0_ = C_Hb_ × [Hb]	
		=1.36 mlO_2_/g × 0.43 g/mL	
		=0.5 (mlO_2_ ml^−1^)	
Tissue O_2_ solubility	a_T_	2.8 × 10^−5^ (mlO_2_ ml^−1^ mmHg^−1^)	Welter 2016
Tissue O_2_ diffusivity	D_T_	2.41 × 10^−9^ (m^2^s^−1^)	Welter 2016
Capillary length	L	0.001 (m)	Less 1991
Capillary radius	R_c_	3.5 × 10^−6^ (m)	Less 1991
Capillary velocity	v	0.00079 (m s^−1^)	Ivanov 1981

The chosen “tissue types” to simulate were: healthy brain tissue, and tumor regions of “vascular periphery,” “necrotic tissue,” “tissue with high metabolism” to simulate hypoxic regions (split into “more hypoxic” and “less hypoxic” by higher and lower blood volume ranges), and “tissue with normal metabolism” to simulate the regions of the tumor which are not hypoxic. To select ranges for OEF, P_crit_, and blood volume that have already been approved in peer‐review were used, such as the values published in a computational model for tumor oxygenation by Welter et al. were used,^25^ or published values that were measured experimentally. For OEF: Welter et al. report an OEF in healthy breast tissue of 0.11 ± 0.09, and breast tumor tissue of 0.34 ± 0.1; Cho et al.^35^ report an OEF in healthy brain tissue of 34.2 ± 2.6%; and necrotic tissue was assumed to be very low, between 0.02 and 0.05. For blood volume: Leenders et al. measured the blood volume in healthy brain tissue to be 5.2% ± 1.4% and 2.7 ± 0.6% for gray and white matter, respectively^36^; Welter et al. report that using MRI and a brain tumor animal model, BV of tumor tissue was 5.3% ± 0.6% which is used as the “low blood volume range,” and Qi et al.^37^ measured 13% ± 4.1% blood volume in VX2 squamous cell tumors, which is used as the “high blood volume” range; for necrotic tissue, a 0.01% blood volume is used, i.e., almost no blood compartment is present. Last, Welter et al. report that literature values of P_crit_ range from 1‐4 mmHg, and Welter et al. lowered P_crit_ for tumor tissue by ½ due to resistance to hypoxia; this corresponds with reports from Honig and Gayeski, who report tumor P_crit_ as 0.5 mmHg.

For the dependent variables, the resulting range of R_t_ calculated from the range of BV values chosen in Table 1 is 1.07–4.43 × 10^−5^ m, which is similar to ranges reported such as 4 × 10^−5^ m^38^ and 2.5 × 10^−5^ m.^39^ Likewise, the resulting range of M_0_ calculated from the range of OEF chosen in Table 2 is 1.2 × 10^−5^–3.5 × 10^−4^, which is consistent with the reported M_0_ values of 6 × 10^−5^ and 2.4 × 10^−4^ mlO_2_/mL/s for normal tissue and tumor tissue, respectively, used by Welter et al.^25^

#### 3.1.2 Comparison with empirical OE‐MRI results

Empirical OE‐MRI measurements by Winter et al.,^10^ Bhogal et al.,^20^ O'Connor et al.,^11^ Little et al.,^8^ and Muir et al.^21^ were used for comparison because they either estimated or measured the PaO_2_ change induced by their hyperoxic gas challenge. Altogether, these studies provided measurements in “healthy brain tissue,”^20^ “vascular periphery,” “tumor core,” “necrotic tissue,”^10^ and whole tumor region of interest (ROI) measurements from clinical patient data by O'Connor et al.,^11^ and Little et al.^8^ As it is not possible to know the composition of the whole tumor ROIs reported, the simulated ΔR1 for each possible tumor tissue type is calculated for comparison, and it is assumed that the empirical whole tumor ROI should fall within that range of ΔR1.

In the measurements from Winter et al., the exact definition of the “necrotic” versus “central tumor” regions is defined as follows^10^: the necrotic core was characterized as exhibiting hypo‐intensity on post‐contrast gadolinium‐enhanced T1‐weighted imaging, suggesting it was avascular, and the necrotic nature of this region was confirmed by histology. In contrast, the “central tumor” was defined as tumor tissue excluding the enhancing vascular rim and excluding necrotic tissue, if any.

## 4 RESULTS

**FIGURE 5:**
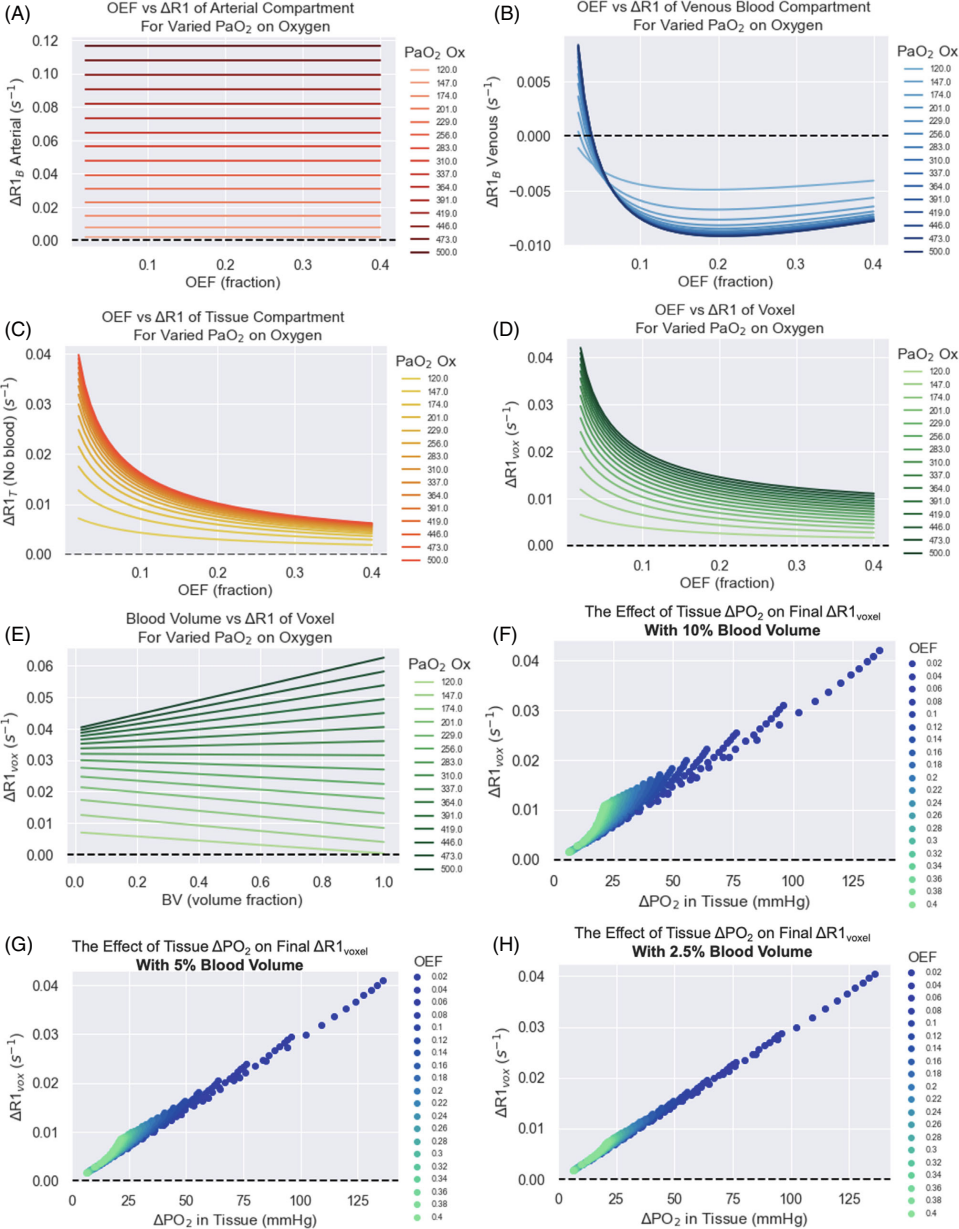
Simulated data showing the effect of OEF on the ΔR1 of the arterial blood (A), venous blood (B), and tissue compartments individually (C), and total voxel ΔR1 (D) for varied PaO_2_ changes induced from increased oxygen breathing, with blood volume set to 10%. E, The corresponding effect of varying blood volume with a set OEF (OEF = 0.2) is shown. F–H, Simulated data showing the relationship between ΔPO_2_ in the tissue compartment and ΔR1_voxel_, for 10% (F), 5% (G), and 2.5% (H) blood volume. The data points are colored by the OEF (0.02–0.40) used in that ΔR1_voxel_ calculation (see OEF legend). All simulations in this figure used B0 = 1.5T

To visualize the relationship between the independent variables, dependent variables, and ΔR1, the following plots were produced. The effect of all combinations of blood volume and OEF on the baseline tissue PO_2_ (mean and minimum) and maximum oxygen consumption rate is shown in Supporting Information Figure S6. The effect of OEF on the ΔR1 of the venous, arterial, and tissue compartments individually, for varied PaO_2_ changes induced from oxygen, is shown in Figure 5A–C, and the resulting ΔR1 for the voxel is shown in Figure 5D,E (all simulated at 1.5T). In this model, there is an inverse mathematical relationship between blood volume and the Krogh Radius R_t_ (shown in Supporting Information Figure S3), and therefore blood volume has an effect on the M_0_ (see Supporting Information Figure S6C), where M_0_ increases with blood volume fraction. In our model, blood volume is considered to be the independent variable while M_0_ is dependent, however in biological reality, this is a highly intertwined feedback relationship where tissues with high metabolism recruit more blood vessels.^40^ Blood volume also has an effect on the final ΔR1_voxel_ (see Figure 5D,E), which is due to the increased contributions from the arterial and venous components in Step 4 of the model. Interestingly, when there has been a smaller change in PaO_2_ (i.e., less oxygen administered), the increase in the blood volume fraction allows the negative ΔR1 from the venous component to decrease the ΔR1 of the voxel, whereas this becomes dominated by the high increase in ΔR1 from the arterial component as more oxygen is administered.

In this model, the OEF chosen has a large effect on ΔR1_voxel_, which can be seen clearly in Figure 5D,E. Figure 5A,E shows that as OEF increases – in other words, as a higher fraction of oxygen is extracted from the tissue between the arterial and venous ends – there will be a lower change in PO_2_ in the tissue (and hence smaller ΔR1_T_), which is the largest contributor to the overall ΔR1 of the voxel. Last, the relationship between ΔPO_2_ in the tissue compartment and ΔR1_voxel_ is shown in Figure 5F‐H.

**FIGURE 6:**
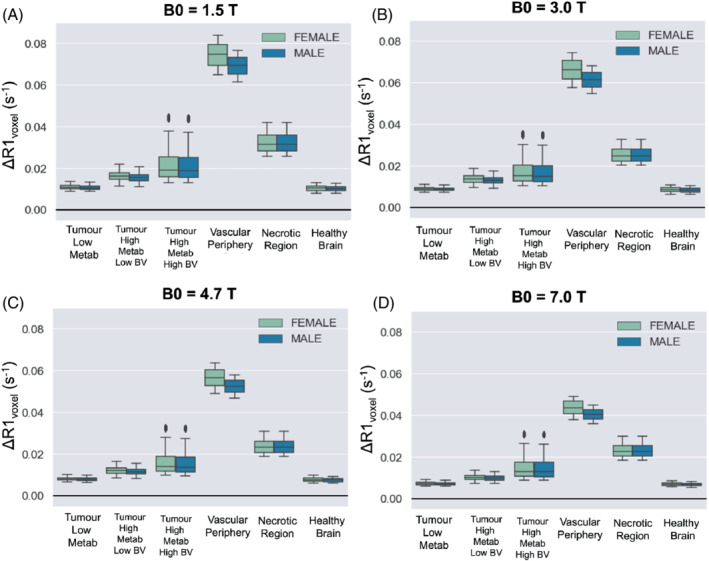
The resulting ΔR1 from a hyperoxic gas challenge calculated from this model, calculated using all combinations of the chosen range of Hct (using the female and male ranges for hematocrit separately), OEF, BV, P_crit_ listed in Table 1 for each respective tumor tissue type, with B0 set to 1.5T (A), 3T (B), 4.7T (C), 7T (D)

The resulting ΔR1 calculated from this model is plotted in Figure 6, calculated using different field strengths, and using all combinations of the chosen range of Hct, OEF, BV, P_crit_ listed in Table 1 for each respective tumor tissue type. Since hematocrit levels can vary between sexes, the results from using the female and male ranges for hematocrit have been shown separately to examine the expected difference in ΔR1. In this simulation, the largest difference in ΔR1 between the sexes was 0.0045 s^−1^, which occurred in the vascular periphery due to containing the highest percentage of blood component and therefore most affected by differences in hematocrit. The difference in ΔR1 between the sexes was negligible in the other simulated tissue types.

**FIGURE 7:**
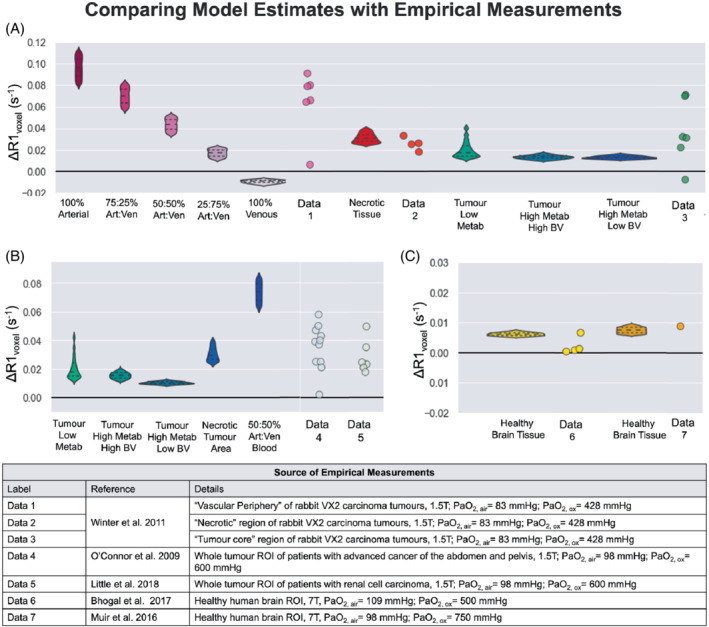
Plots containing seven sets of empirical ΔR1 measurements, alongside the respective simulated ΔR1 according to the B0, PaO_2_, and tissue type

For a rough quantitative comparison, empirical _ΔR1_ measurements from six tissue types, alongside the respective simulated ΔR1 according to the B0, PaO_2_ and tissue type, are shown in Figure 7. It is not possible to know the composition of the whole tumors ROIs reported by O'Connor et al.^11^ and Little et al.,^8^ or the “tumor core” reported by Winter et al.,^10^ however, each data point for empirical tumor ΔR1 falls within the range of ΔR1 simulated for each possible tumor tissue type; therefore, these whole tumor ROIs could reasonably be the sum of a composition of these different tumor tissue types. One notable exception to this is one “tumor core” data point reported by Winter et al.,^10^ which reaches a negative ΔR1 close to that of pure venous blood.

## 5 DISCUSSION

In this paper, we propose a three‐compartment model for estimating the changes in R1 that could be expected in tumor tissues depending on field strength, blood SO_2_, blood volume, hematocrit, oxygen extraction fraction, and changes in PO_2_ in both the blood and tissue. This model has been developed with the aim of estimating the expected ΔR1 induced by the oxygen delivery in a voxel containing tumor tissue, however, it is generally applicable to OE‐MRI research as well and has been designed to make it possible for a researcher to easily substitute their preferred model for tissue oxygen diffusion and consumption and make this model tailored to their tissue of interest, for example, placenta or liver.

Interestingly, the relationship between ΔPO_2_ in the tissue compartment and ΔR1_voxel_ (Figure 5F‐H) shows that the linear relationship between ΔPO_2_ and ΔR1_voxel_ seen in phantoms almost holds true in tissue voxels containing lower blood volume; however, in voxels containing higher blood volume, the ΔR1 contribution from the blood compartment interrupts the linear relationship. This suggests that despite the influence of deoxyhemoglobin changes, measuring ΔR1 does provide an indication of the final change in PO_2_ in the tissue. In practice, the efficiency of the oxygen delivery to the tissue via inhalation of increased oxygen fraction can be affected by by many factors, and therefore for convenience, Figure 5D simulated a large range of PaO_2_ changes (120‐500 mmHg) where the resulting change in the R1 (at 1.5T) of the voxel can be seen for each respective PaO_2_. In addition, Figures 5F‐H display the estimated corresponding ΔR1 for a large range of levels of changes in tissue PO_2_ at 1.5T, allowing for a convenient estimate of the corresponding ΔR1 by quickly viewing the data points in Figure 5F‐H.

It is known that the values estimated by the r1_Ox_ model and R1 Blood models by Bluemke et al.^15^, ^19^ both agree well with empirical measurements (R^2^ = 0.93 and 0.93); however, since the tissue compartment of this model contains variables that were not measured at the time of OE‐MRI data collection (i.e., hematocrit, changes in arterial PO_2_, tumor blood volume), it is not possible to quantitatively compare the model ΔR1 predictions to the measured ΔR1 with metrics such as R^2^ and MSE. Instead, we used a variety of reported ΔR1 from OE‐MRI literature to gain a rough estimation of the accuracy of this model: qualitative ΔR1 responses, categorized by different tumor tissue types by the authors of the OE‐MRI literature, are listed in Supporting Information Table S1. Overall, the simulated ΔR1 for each tissue type shown in Figure 6 does correspond with observations from OE‐MRI literature: as seen in experiments by Winter et al.,^10^ the “vascular periphery” shows greater +ΔR1 than the “tumor core” and “necrotic” regions, and the “necrotic” regions show a greater +ΔR1 than the “tumor core” but less than “vascular periphery.” This is consistent with the distinctions between these three regions defined by Winter et al. — (1) necrotic region is avascular and filled with fluid, (2) the central tumor is cell‐dense and has much lower vascularity than vascular periphery. As observed in experiments by Burrell et al.,^41^ the “less hypoxic” tumor type shows greater +ΔR1 than the “more hypoxic” tumor type. Last, in these simulations, the healthy brain tissue is predicted to show a very small ΔR1 that might end up ‘not detectable’, as observed by Bhogal et al.^20^

For a select few OE‐MRI studies that did report either measured or estimated PaO_2_ changes, the simulated ΔR1 from the model did result in ΔR1 values that were in good agreement with the empirical data (Figure 7). The values for brain tissue and necrotic tissue were particularly accurate, and although it Is not possible to know the composition of the whole tumors ROIs, “vascular periphery,” or “tumor core,” each data point for empirical tumor ΔR1 fell within the range of ΔR1 simulated for each possible tumor tissue type. Therefore, the empirical ΔR1 could reasonably be the sum of a composition of these different tumor tissue types.

This model may be useful for OE‐MRI researchers looking to predict the effect of certain factors, such as hematocrit. It is interesting that hematocrit differences in the male and female populations did have a slight effect on ΔR1 when the voxel contained larger blood volumes. In fact, this predicted difference has been empirically observed in lung tissue — where blood volume is approximately 33%–36%^42^ — Kindvall et al.^12^ reported that age and sex were all predictors of ΔR1 in lung tissue, likely due to the hematocrit differences.

### 5.1 Limitations

It is currently difficult to quantify the agreement of this model with the empirical data, and the model still includes some constants and variables that cannot be measured in individual patients, although it has been designed so that the independent variables are either known (such as B0) or can be measured via a non‐imaging method (i.e., arterial PO_2_, hematocrit), or through another imaging method (i.e., measuring blood volume or OEF). Testing whether it would be possible to use OEF or blood volume measurements from another imaging modality is beyond the scope of this paper, however, in the future, as methods for measuring blood volume or OEF continue to advance, this connection could prove useful in future work. In the future, it is possible that combining R1 measurements with other MRI techniques such as R2* or oxygen‐17 gas^43^ could improve the accuracy and robustness for monitoring oxygenation.

In addition, there may be unpublished raw datasets from former OE‐MRI studies held by other research groups where arterial PO_2_ was measured but perhaps not reported. We welcome other researchers to test the predictions of this model against a larger sample size of data — this would greatly improve confidence in this model before it is applied in oxygen‐enhanced MRI studies.

Another limitation is that, in this current model, the arterial blood volume has been set to be equal to the venous blood volume — this will not be true for all voxels. Since we have provided the open‐source code for the model, future researchers are welcome to adjust this parameter if they have more information about the ratio of arterial and venous blood in their voxels of interest.

The the parameters for the r1_Ox_ model by Bluemke et al.^15^ were created by compiling empirical measurements of the relaxivity of oxygen over 50 y of MRI research in phantoms, saline and water solutions, vitreous fluid, and plasma, and fitting these data to a Lorentzian equation that fit R^2^ = 0.93. Of course, the r1_Ox_ in these solutions may be slightly different than the r1_Ox_ in tissues. Indeed, the r1_Ox_ may vary between tissues as well. However, we believe the r1_Ox_ derived from this empirically driven model will better represent the r1_Ox_ in tissue than using any single r1_Ox_ datapoint measured in saline or water, as has been common practice in previously published work using r1_Ox_ values for various calculations.^3^, ^5^, ^14^, ^22^, ^23^, ^44^, ^45^

This model contains fewer independent variables than the model by Holliday et al.,^22^ but considerably more variables than the simple equation proposed by Kindvall et al.^23^ Ideally, the outputs of these three models could be compared; however, since they are so different and contain such different parameters, it is difficult to choose the variable ”settings” at which to compare them. For example, this new model adjusts all field‐dependent parameters according to field strength, while both of the previous models are only applicable at one field strength. Similarly, neither of the previous models account for hematocrit differences, which *do* affect the resulting ΔR1, as we have now demonstrated in this manuscript and as was actually measured in human lung tissue by Kindvall et al.^23^ Therefore, although it is not possible to provide a robust comparison of this model to the previous two models, we are confident that this model brings significant improvements for two main reasons: first, the utility of this model surpasses the previous models simply by the fact that the previous models only apply to a single field strength.^22^, ^23^ OE‐MRI research occurs at a variety of field strengths, and therefore the model must account for the effect of B0 on each of the relevant variables. This field‐strength consideration is an extremely useful feature that will allow data acquired at different field strengths to be compared. Second, one previous model incorrectly combines values from different field strengths into one single model (using r1_dHb_ from Silvennoinnen et al.^24^ at 4.7T alongside r1_Ox_ from Pilkinton et al.^46^ at 1.5T), which suggests it will be produce slightly incorrect results at any field strength.^22^ In summary, we present a new model that is more accurate and considers important factors such as field strength and hematocrit. Most of all, however, our work extends the previous modeling work in significant ways, in particular by incorporating the concept of OEF and introducing an alternative approach to incorporating metabolic rate.

Last, a number of assumptions are necessarily made in the generation of this model, however one may be particularly problematic for some research applications: the assumption from the Krogh tissue model that all capillaries are parallel, unbranched, and equally spaced. While this may be a reasonable assumption in the brain and certain other organs, in others it is a very poor assumption. i.e. the placenta, where OE‐MRI has been used successfully.^47^ Importantly, this is also a very poor assumption in tumors, which often have severely deranged vasculature, including tortuous and elongated capillaries.^48^ This is a major limitation of using the Krogh model to estimate the tissue PO_2_ changes, as deviations from the simplistic geometry assumed may cause misinterpretations. For example, complex geometry will affect the ability to estimate R_t_, which will then cause errors in the modeled metabolic rate. Fortunately, the modular nature of this model and the supplied code allows researchers to easily substitute more modern tissue oxygen diffusion and consumption models that do account for abnormal vasculature, or any other particular qualities that their tissue of interest may require.

## 6 CONCLUSIONS

In conclusion, we have proposed a three‐compartment model for estimating the changes in R1 that could be expected in various tissues depending on field strength B0, SO_2_, BV, hematocrit, oxygen extraction fraction (OEF), and changes in blood and tissue PO_2_. In a demonstration of the model, the resulting ΔR1 are consistent with reported literature OE‐MRI results in a variety of tissues. This model has been designed to be easy for researchers to tailor to their tissue of interest by substituting their preferred model for tissue oxygen diffusion and consumption.

## Supporting information


**Figure S1:** The (A) total oxygen content (CaO_2_), (B) SO_2_, and (C) PO_2_ along the capillary for OEF ranging from 0.2–0.4
**Figure S2:** (A) The behavior of the oxygen consumption rate (M) as described by the Michaelis–Menten model (Equation 10) for a range of values chosen for P_crit_ (0.5‐4 mmHg). (B) The behavior of PO_2_ into the tissue radius as described by the Krogh‐Erlang solution (Equation 9), is shown for a range of M_0_ to illustrate the effect of different rates of M_0_ on PO_2_ (with constant P_cap_ = 90 mmHg).
**Figure S3:** (A) An illustration of a 3D voxel of tissue with capillaries as cylinders that are parallel, unbranched, and equally spaced throughout the voxel. (B) The inverse relationship between blood volume and the Krogh Radius R_t_ resulting from following this method
**Figure S4:** Example 3D surface plots (shown from two viewing angles) of the PO_2_ into the tissue radius and along the capillary, on (A) air and (B) oxygen breathing, assuming a post‐oxygen arterial PO_2_ change from 90‐200mHg.
**Figure S5:** A schematic of Step 4, where the ΔR1 from each compartment is weighted by the volume fraction of each compartment within the voxel. The final ΔR1_voxel_ is the sum of the weighted components
**Figure S6:** Simulated data showing the resulting (A) mean tissue PO_2_, (B) minimum tissue PO_2_, and (C) maximum oxygen consumption rate resulting from all combinations of the ranges of blood volume and OEF used. (D) The blood volume vs. mean tissue PO_2_ is shown for just OEF = 0.4, to show the subtle curve not visible in plots A‐B due to the scale of the y‐axis. All plots use PaO_2_ = 90 mmHg
**Table S1:** The OE‐MRI response in different tissue types identified by various studies

## Data Availability

The model has been hosted open‐source at [github.com/BulteGroup/TissueR1Model] for other researchers to adopt, adapt and improve.
