# Supplementary material for: Modeling the Effect of Hyperoxia on the Spin–Lattice Relaxation Rate R1 of Tissues
Source: Magn Reson Med. 2022 Jun 9;88(4):1867–85. doi: 10.1002/mrm.29315 (PMC9545427; doi:10.1002/mrm.29315)
Supplement: Supplementary file 1 — Figure S1: The (A) total oxygen content (CaO2), (B) SO2, and (C) PO2 along the capillary for OEF ranging from 0.2–0.4 Figure S2: (A) The behavior of the oxygen consumption rate (M) as described by the Michaelis–Menten model (Equation 10) for a range of values chosen for Pcrit (0.5‐4 mmHg). (B) The behavior of PO2 into the tissue radius as described by the Krogh‐Erlang solution (Equation 9), is shown for a range of M0 to illustrate the effect of different rates of M0 on PO2 (with constant Pcap = 90 mmHg). Figure S3: (A) An illustration of a 3D voxel of tissue with capillaries as cylinders that are parallel, unbranched, and equally spaced throughout the voxel. (B) The inverse relationship between blood volume and the Krogh Radius Rt resulting from following this method Figure S4: Example 3D surface plots (shown from two viewing angles) of the PO2 into the tissue radius and along the capillary, on (A) air and (B) oxygen breathing, assuming a post‐oxygen arterial PO2 change from 90‐200mHg. Figure S5: A schematic of Step 4, where the ΔR1 from each compartment is weighted by the volume fraction of each compartment within the voxel. The final ΔR1voxel is the sum of the weighted components Figure S6: Simulated data showing the resulting (A) mean tissue PO2, (B) minimum tissue PO2, and (C) maximum oxygen consumption rate resulting from all combinations of the ranges of blood volume and OEF used. (D) The blood volume vs. mean tissue PO2 is shown for just OEF = 0.4, to show the subtle curve not visible in plots A‐B due to the scale of the y‐axis. All plots use PaO2 = 90 mmHg Table S1: The OE‐MRI response in different tissue types identified by various studies [file MRM-88-1867-s001.docx]

# Supporting Information

## *Supporting Information Figures*


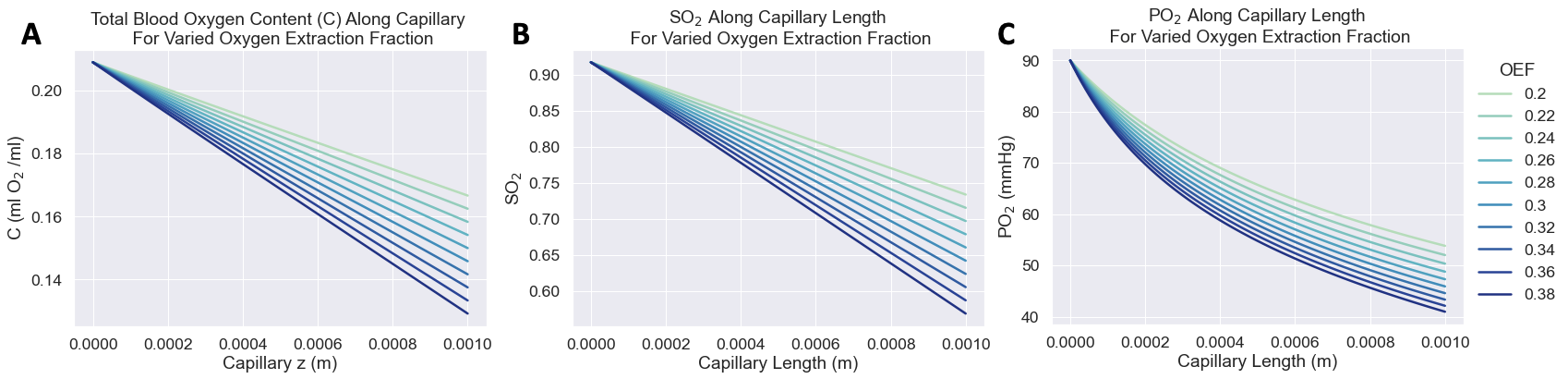


##### Supporting Information Figure S1: The (A) total oxygen content (CaO_2_), (B) SO_2_, and (C) PO_2_ along the capillary for OEF ranging from 0.2-0.4.

#####
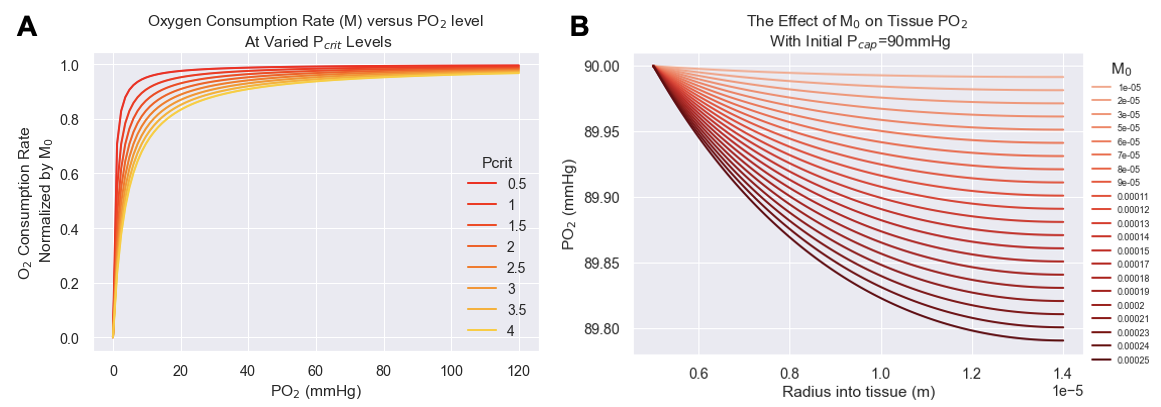
Supporting Information Figure S2: (A) The behaviour of the oxygen consumption rate (M) as described by the Michaelis-Menten model (Equation 10) for a range of values chosen for P_crit_ (0.5-4mmHg). (B) The behaviour of PO_2_ into the tissue radius as described by the Krogh-Erlang solution (Equation 9), is shown for a range of M_0_ to illustrate the effect of different rates of M_0_ on PO_2_ (with constant P_cap_= 90 mmHg).


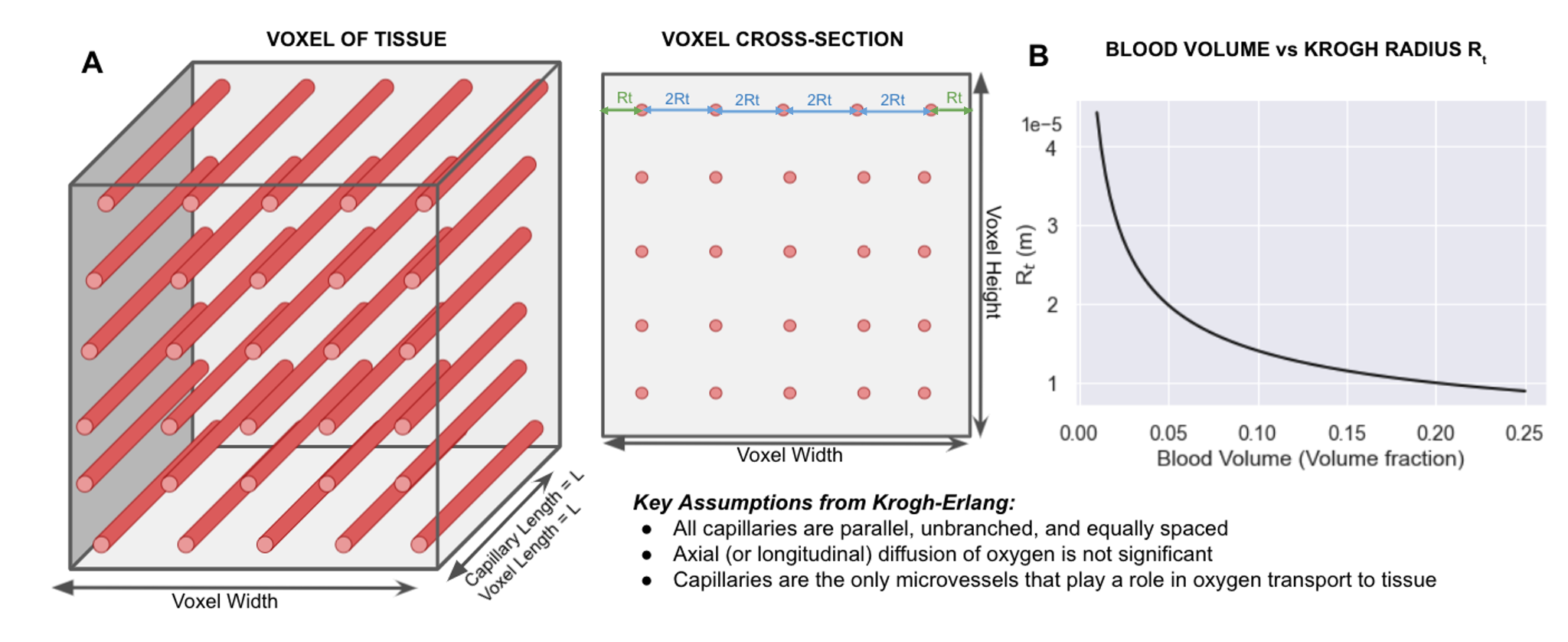


##### Supporting Information Figure S3: (A) An illustration of a 3D voxel of tissue with capillaries as cylinders that are parallel, unbranched, and equally spaced throughout the voxel. (B) The inverse relationship between blood volume and the Krogh Radius R_t_ resulting from following this method.


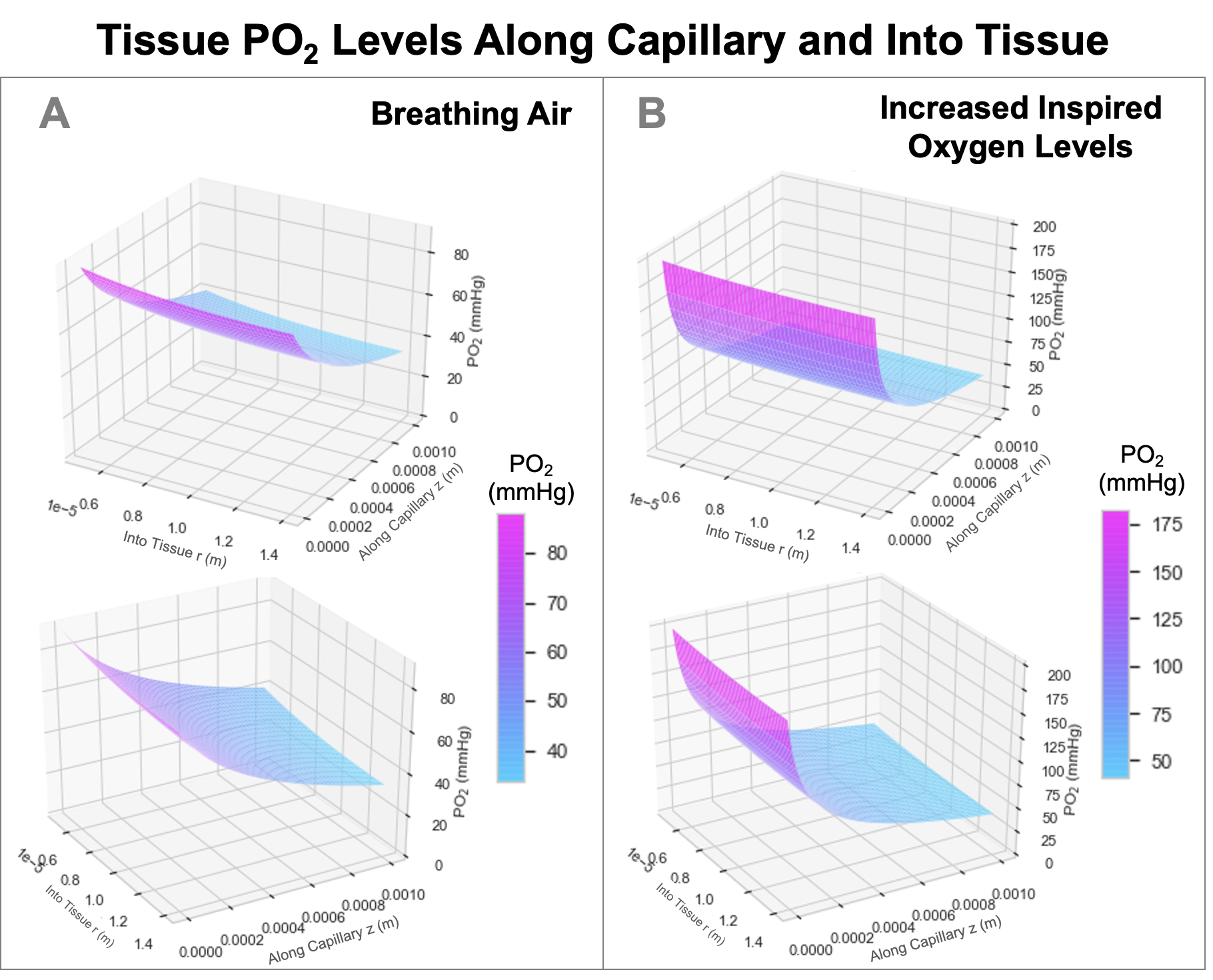


##### Supporting Information Figure S4: Example 3D surface plots (shown from two viewing angles) of the PO_2_ into the tissue radius and along the capillary, on (A) air and (B) oxygen breathing, assuming a post-oxygen arterial PO_2_ change from 90-200mHg.


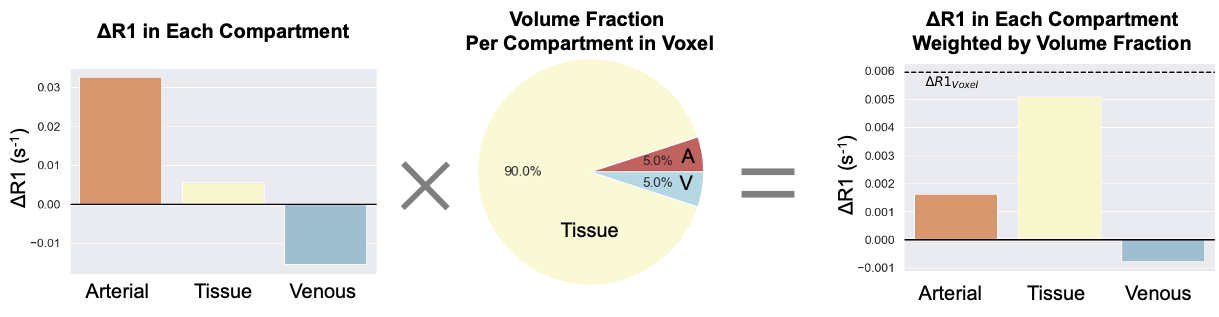


##### Supporting Information Figure S5: A schematic of Step 4, where the ΔR1 from each compartment is weighted by the volume fraction of each compartment within the voxel. The final ΔR1_voxel_ is the sum of the weighted components.


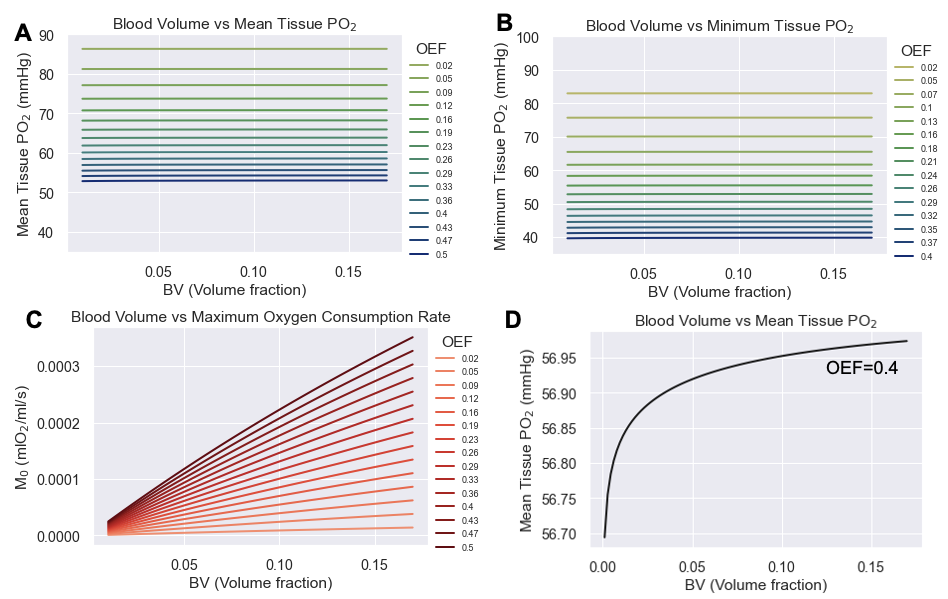


##### Supporting Information Figure S6: Simulated data showing the resulting (A) mean tissue PO_2_, (B) minimum tissue PO_2_, and (C) maximum oxygen consumption rate resulting from all combinations of the ranges of blood volume and OEF used. (D) The blood volume vs. mean tissue PO_2_ is shown for just OEF=0.4, to show the subtle curve not visible in plots A-B due to the scale of the y-axis. All plots use PaO_2_=90mmHg.

##

## *Supporting Information Tables*

##### Supporting Information Table S1: The OE-MRI response in different tissue types identified by various studies.

| **Generalized Tumour Tissue Type** | **Empirical ΔR1 Response to 100% O2** |
| --- | --- |
| Well-perfused tissues, vascular rim tissue, or called “Vascular Periphery” | - Positive ΔR1 (1.5T) (O’Connor et al., 2009) - Positive ΔR1 (1.5T) (Winter et al., 2011) |
| Tumour core, possibly hypoxic, but is perfused, or hypoxic region that consumes the O_2_ | - Mostly positive ΔR1 (1 small negative ΔR1) (1.5T) (Winter et al., 2011) - Low or absent ΔR1, but moderate perfusion from IAUC60 (1.5T) (O’Connor et al., 2009) - More hypoxic tumour type showed smaller positive ΔR1, less hypoxic tumour type showed larger positive ΔR1* (7T) (Burrell et al., 2013) (*Used carbogen) - Strong negative ΔR1 seen in the contrast enhancing areas of the glioblastoma and metastasis (1.5) (Remmele et al., 2013) |
| Necrotic/avascular tissue that has little perfusion | - Positive ΔR1 (1.5T) (Winter et al., 2011) - Moderate positive ΔR1 in peritumoral edema and necrotic areas (1.5) (Remmele et al., 2013) - Low or absent ΔR1 (1.5T) (O’Connor et al., 2009) |

# 
